# Preparation and Application of Polyrotaxane Cross-Linking Agent Based on Cyclodextrin in Gel Materials Field

**DOI:** 10.3390/gels9110854

**Published:** 2023-10-28

**Authors:** Siyuan Liu, Jingxi Zheng, Jiaqin Wang, Shanghao Liu, Xianli Zhang, Dan Bao, Peng Zhang

**Affiliations:** 1 School of Chemistry and Chemical Engineering, Chongqing University of Science and Technology, Chongqing 401331, China; 2021205180@cqust.edu.cn (S.L.); zhengjingxi2023@163.com (J.Z.); 2022205018@cqust.edu.cn (J.W.); 2022205066@cqust.edu.cn (S.L.); 2021205173@cqust.edu.cn (X.Z.); 18765920408@163.com (D.B.); 2 Chongqing Oil and Gas Chemical Engineering Technology Research Center, Chongqing 401331, China

**Keywords:** cyclodextrin, polyrotaxane cross-linking agent, preparation, application, “slide ring” structure

## Abstract

The cross-linking point of a conventional chemical cross-linking agent is fixed. Therefore, gels that are prepared with a conventional cross-linking agent have poor deformability, strength, shear resistance, and further properties. Some researchers have prepared a new cross-linking agent using cyclodextrin (CD). In a polyrotaxane cross-linking agent, the cross-linking points can slide freely along the molecule chain. The special “slide ring” structure can provide better elongation, strength, and other properties to gels, which can effectively expand the application of the gel’s materials. This paper summarizes the preparation methods and applications from different types of CD and compares the improvements of properties (swelling, viscoelastic properties, etc.). In addition, the current results of our group are presented, and some ideas are provided for the development of polyrotaxane cross-linking agents.

## 1. Introduction

Since the discovery of crown ethers by Pedersen et al. [1] in 1967, mankind opened the field of supramolecule chemistry. Supramolecular chemistry refers to two or more molecules or ionic compounds through intermolecular interactions [2,3,4,5,6], which endow materials with some unique properties that differ from conventional materials. Among those intermolecular interactions, host–guest chemistry is a highly promising direction. The host chemical substances can select the guest chemical substances based on some special molecular forces, and the guest chemical substances need to meet some special structures to form stable structures with the host chemical substances, just like a matching of key and lock.

**Figure 1:**
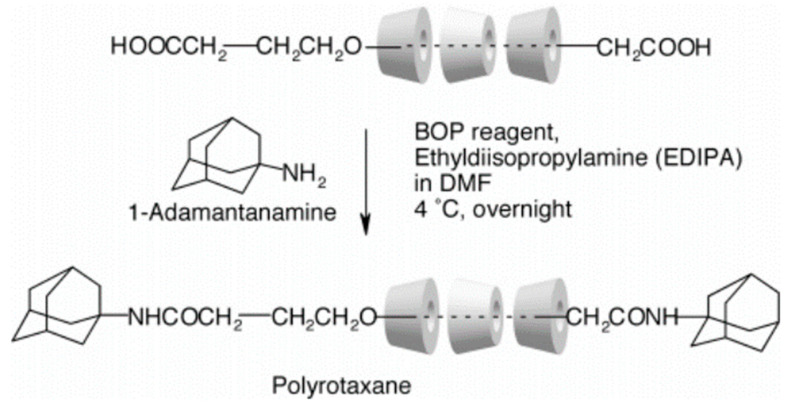
Schematic diagram of the plugging the extremities [11].

**Figure 2:**
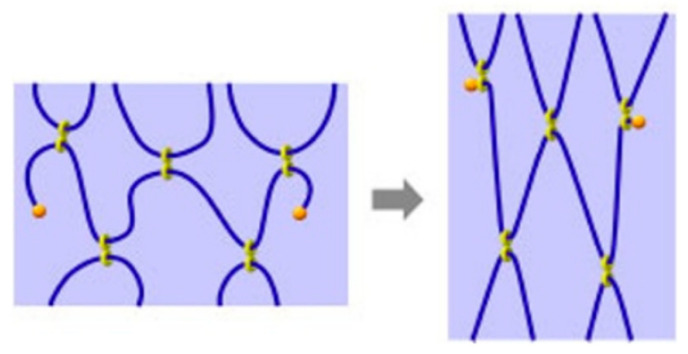
Schematic diagram of “slide-ring” cross-linking points [14].

Owing to the unique outer hydrophilic and inner lipophilic structure of cyclodextrin (CD), polymer chains with specific structures can pass through the cavities to form a stable encapsulated structure. It has attracted widespread attention from researchers [7,8,9]. In 1990, Harada et al. [7] used polyethylene glycol (PEG) to prepare pseudopolyrotaxane by passing through the cavity of α-CD, a ring-like molecule that can slide freely on a long-chain molecule, known as a “slide ring” structure. To prevent the CDs from sliding from the long-chain molecule, some researchers use bulky molecules such as *1*-adamantanamine, dinitrobenzene, etc., to plug the long-chain molecule [10,11,12,13], as shown in Figure 1. Two CDs coupled together with shorter or longer spacer form “8” structure, where each cavity can contain different polymer chains. The two CDs behave as crosslinking points, giving high flexibility to the polymer chains, as shown in Figure 2 [14]. Conventional gel materials have poor shear resistance, strength, and deformation properties. Some researchers prepared new gel materials to overcome those disadvantages [15,16,17,18].

To prepare a new gel, some researchers try to introduce a “slide-ring” structure into polymers. The new gel can overcome the disadvantages of conventional gels and has better properties in deformability, strength, and self-healing properties. However, the wide use of the new gel material is limited. On the other hand, plugging the extremities is the necessary operation because of the intermolecular force [19], or CDs may fall out from the long-chain molecule, resulting in the “slide-ring” structure being destroyed. On the other hand, the types of long-chain molecule and CD also affect the preparation of the new gel material. The inner diameters of the α-CD, β-CD, and γ-CD are 0.50 nm, 0.62 nm, and 0.80 nm, respectively [20]. Influenced by intermolecular forces, cavity size, and other factors, only a small number of long-chain molecules can pass through the cavity to form a “slide-ring” structure, including polydiols [21,22], polyolefins [23,24], and polyethers.

Based on the above, some researchers propose modifying the hydroxyl groups on CDs to prepare the cross-linking agent, which can effectively reduce the steps of preparation and improve productivity. Compared with conventional chemical cross-linking agents, such as polyethylene glycol diacrylate (PEGDA), *N*, *N*-methylene bisacrylamide (BIS), etc., the polyrotaxane cross-linking agent can effectively improve the deformity and strength of gels materials based on the “slide-ring” structure. This paper summarizes the preparation and properties of the polyrotaxane cross-linking agent based on different types of CD.

## 2. The Preparation and Application of Hydrogel Based on α-CD Cross-Linking Agent

**Figure 3:**
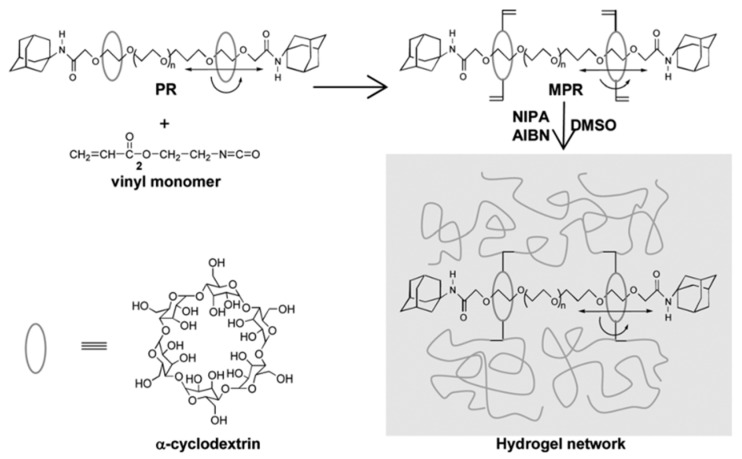
Schematic representation of the preparation of the movable cross-linking agent and its gelation [25].

**Figure 4:**
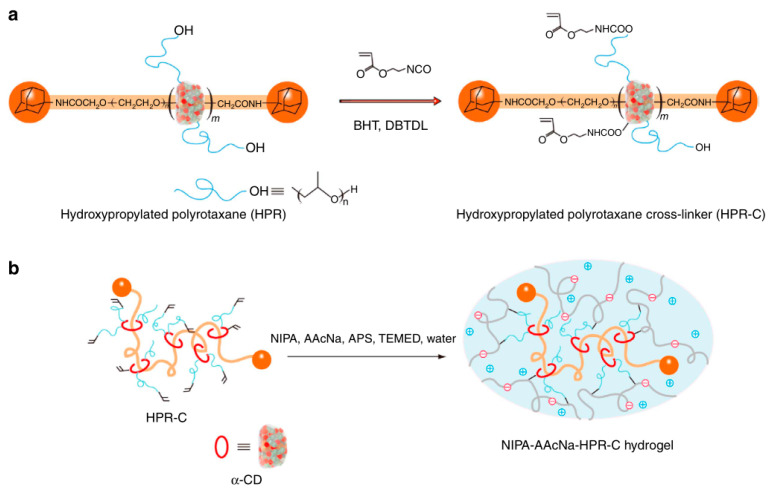
(**a**,**b**) Preparation of the polyelectrolyte hydrogels using nonionic PR cross-linking agent [22].

**Figure 5:**
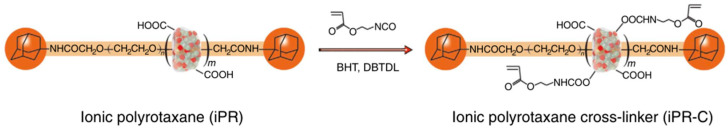
Preparation of the ionic polyrotaxane cross-linking agent based on α-CD and PEG [22].

Some researchers make full use of α-CD to prepare polyrotaxane and then introduced the carbon–carbon double bonds into the polyrotaxane to prepare α-CD polyrotaxane cross-linking agents. A series of polyrotaxane polymer materials with a “slide-ring” effect were prepared based on the above. A limited number of materials were used for hydrogels. The most established system was the PEG chains passing through the cavity of α-CD. Imran et al. [22,25,26,27,28] prepared a hydrophilic polyrotaxane-modified vinyl, which was regarded as the cross-linking agent to prepare a high-strength slide-ring hydrogel. First, α-CD reacted with PEG modified by carboxylic acid groups to obtain α-CD pseudopolyrotaxane. Then, *1*-adamantanamine was employed to plug the extremities of α-CD pseudopolyrotaxane, and polyrotaxane was obtained. Alternatively, propylene oxide has been used to modify the α-CD of polyrotaxane, resulting in the polyrotaxane being hydroxypropylated. Then, the polyrotaxane reacted with the isocyanate ethyl acrylate to obtain the polyrotaxane cross-linking agent. Finally, the polyrotaxane cross-linking agent copolymerized with *N*-isopropylacrylamide (NIPA) to obtain a new high-strength hydrogel or copolymerized with thermosensitive monomers and sodium acrylate to obtain a thermosensitive hydrogel with excellent ductility and toughness that has anionic groups. The preparation process of the hydrogel is shown in Figure 3. According to the strain-controlled dynamic frequency sweep test, the storage modulus was always greater than the loss modulus, indicating that a stable polymer network was formed inside the gel. Furthermore, when the frequency was increased, the gel could equilibrate the tension due to the “slide-ring” effect, which showed the characteristics closer to a liquid [25]. The preparation process of “slide-ring” hydrogel based on α-CD polyrotaxane cross-linking agent is shown in Figure 4. The new hydrogel had high strength and high deformability. The ionic groups could improve the dispersion of the polyrotaxane cross-linking agent in the polymer network. Thus, the introduction of ionic groups into the hydrogel could promote the polymer cross-linked network to synergize with the “slide-ring” cross-linked structure, which could enhance the network extensibility of the polymer and balance the external forces evenly. α-CD and PEG were copolymerized to prepare a new stretchable hydrogel with good toughness as the ionic cross-linking agent, and the preparation process is shown in Figure 5 [22]. The results of rheological testing of the new hydrogel and the conventional hydrogel showed that the swelling rate was similar in water or DMSO-d_6_ solution, and the new hydrogel did not show fractures, whereas fractures were observed in the conventional hydrogel. In addition, small-angle X-ray diffraction (SAXS) was devoted to confirming the structural homogeneity of the new hydrogel, and the result showed that the novel hydrogel remained isotropic.

**Figure 6:**
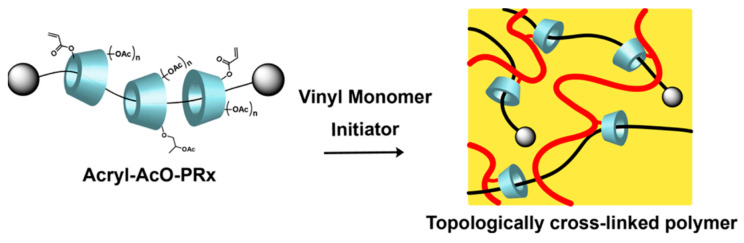
Preparation of topological cross-linked polymers [29].

The idea of Harada’s group [29] was similar to Imran. They are experienced in utilizing α-CD to react with propylene oxide. Then, the carbon–carbon double bonds were introduced by reacting with acryloyl chloride. Finally, modified α-CD reacted with acetic anhydride to obtain α-CD polyrotaxane cross-linking agent. It copolymerized with ethyl acrylate or butyl acrylate to obtain a hydrogel, and the preparation process of the topologically crosslinked polymer is shown in Figure 6. Compared with the stress–strain curve, the strain at break of the new hydrogel material was up to about 600%, which was much higher than the conventional chemical hydrogel material. During the tensile deformation test, the conventional chemical hydrogel broke before reaching plastic deformation, whereas the new hydrogel dispersed the stress through a sliding structure and had an average recovery rate of 74% between healing intervals.

Lin et al. [30] modified both extremities of PEG using *p*-toluenesulfonyl chloride (TsCl) to produce *p*-toluenesulfonylated PEG in the presence of dichloromethane and pyridine. Then, the modified PEG was mixed with α-CD resulting in the chain *p*-toluenesulfonylated PEG passing through the cavities of a plurality of α-CD molecules to form α-CD pseudopolyrotaxane. After that, *3*,*5*-dimethylphenol undergoes a substitution reaction with α-CD pseudopolyrotaxane in a mixed solution system of *N*, *N*′-dimethylformamide and sodium hydride to obtain α-CD polyrotaxanes. The polyrotaxane cross-linking agent based on α-CD derivatives was prepared by reacting α-CD pseudopolyrotaxane with allyl glycidyl ether (AGE) in the solvent dimethyl sulfoxide. Liu [31], Chen [32], and Zhang [33] also successfully prepared α-CD polyrotaxane cross-linking agents using this method. They utilized α-CD polyrotaxane cross-linking agent to modify the permeation vaporization membrane resulting in the permeation vaporization membrane having an excellent separation performance. Due to the special “slide-ring” structure, the distribution of cross-linking points in the permeation vaporization membrane was more uniform, and the mass transfer resistance was greatly reduced.

**Figure 7:**
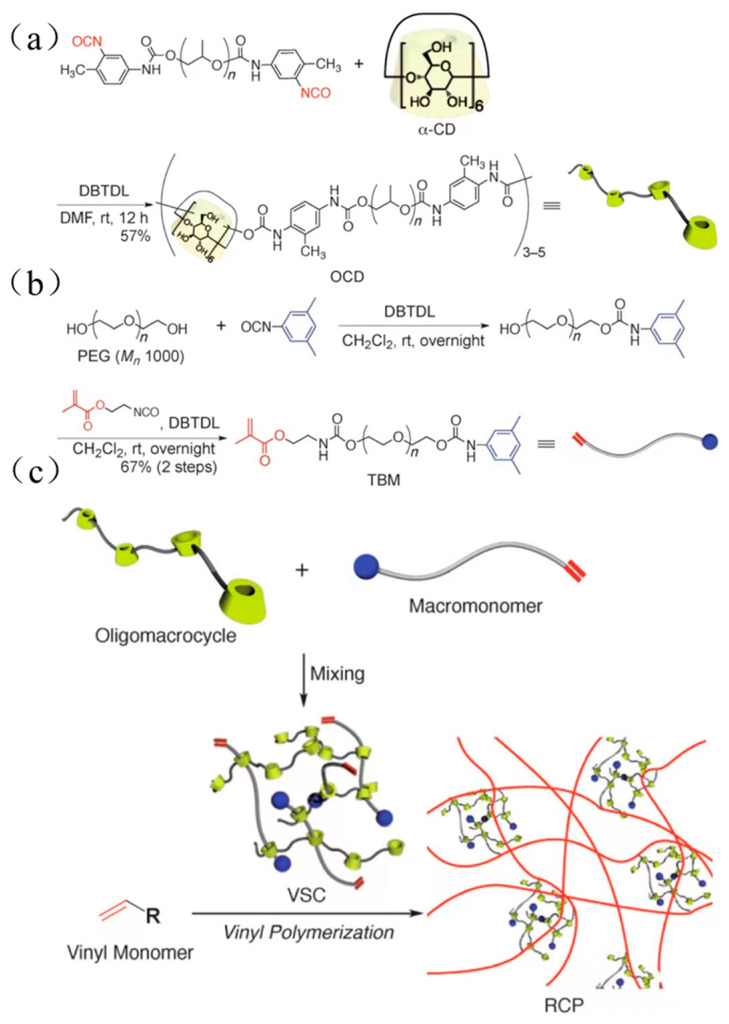
(**a**–**c**) Preparation of α-CD rotaxane cross-linked polymer [34].

**Figure 8:**
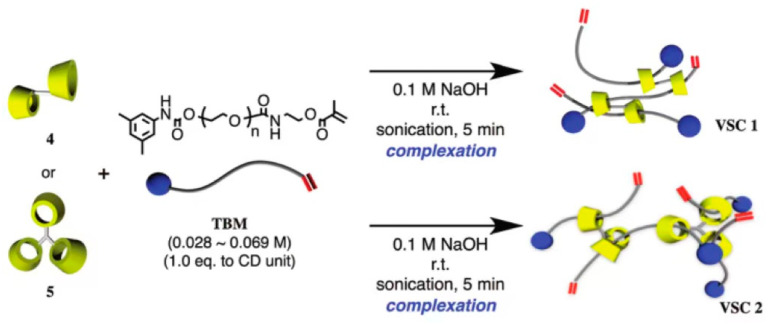
Preparation of rotaxane cross-linking agent by reacting α-CD dimer or trimer with polyethylene glycol type methacrylate [36].

The bulky molecules were regarded as the plugging agent in the previous method. Although it prevented CDs from sliding from the long-chain molecules, the preparation process was cumbersome, and the productivity was low. Some researchers utilized α-CD polymers to react with PEG methacrylates (TBM) to obtain an α-CD polyrotaxane cross-linking agent. One of the extremities was plugged with bulky groups, and another one contained carbon–carbon double bonds, which could react with monomers to plug the extremity through polymerization. Although the method could reduce the preparation steps, it might cause CD to fall out from the long-chain molecule. Arai et al. [34] reported the preparation of a polyrotaxane cross-linking agent, and the process is shown in Figure 7. First, toluene diisocyanate (TDI) plugged with polypropylene glycol reacted with dibutyltin dilaurate (DBTDL) and α-CD to obtain oligomeric CD (OCD). Then, the macromolecular monomers based on hydroxyl-plugged PEG reacted with *5*-dimethylphenyl isocyanate to prepare TBM. Finally, OCD was mixed with TBM, and an α-CD polyrotaxane cross-linking agent (VSC) was obtained. VSC reacted with photoinitiators and vinyl monomers, and then the mixture was degassed and irradiated with UV at room temperature for 3 min to obtain hydrogel materials. Alternatively, VSC reacted with vinyl monomer and degassed, then *N*, *N*, *N*′, *N*′-tetramethylethylenediamine (TMEDA), and potassium persulfate (KPS) were added to the mixture and reacted overnight at room temperature to obtain the hydrogel material. Finally, the hydrogel was dried in an oven at 60 °C for 1 day to purify the rotaxane cross-linked polymer (RCP). The new hydrogel showed a great swelling property (*η_Swelling_* > 1800%) in the different liquid solutions, such as water, methanol, THF, etc. Compared with hydrogel based on polyethylene glycol diacrylate (molecule weight 400) as the cross-linking agent, the elongation at break was improved from 880% to 1210% at a low cross-linking degree and from 310% to 790% at a high cross-linking degree. Sawada et al. [35] reported a rotaxane cross-linking agent based on crown ether and prepared an elastomer with high toughness. A method was reported by Iijima et al. [36] for the preparation of α-CD polyrotaxane cross-linking agent based on α-CD dimers or α-CD trimers, using the reaction of α-CD dimers or α-CD trimers with TBM to obtain VSCs, and the preparation process is shown in Figure 8.

**Figure 9:**
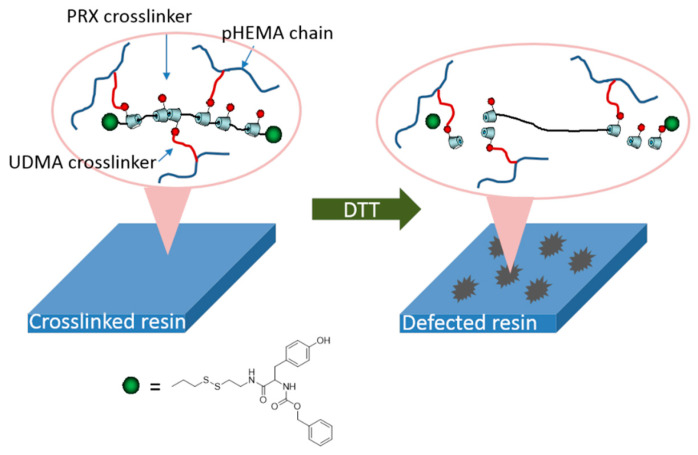
Schematic diagram of the process of the degradable polyrotaxane cross-linking agent [37].

**Figure 10:**
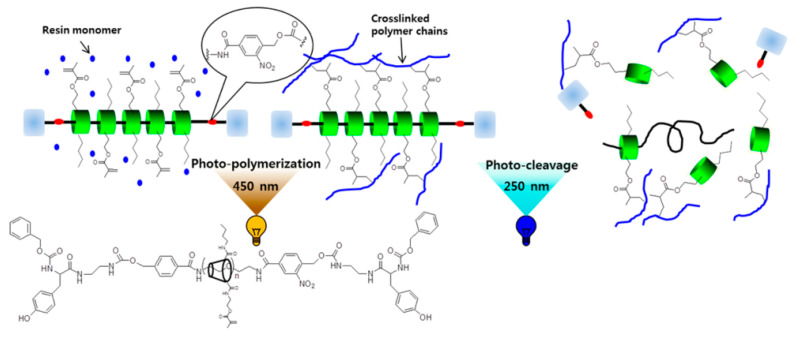
Preparation of UV-Cleavable Photocurable Resin Plastics [38].

The materials synthesized from α-CD polyrotaxane cross-linking agent prepared by the above two methods had a “slide-ring” effect. Seo et al. [37] reported a photocurable plastic based on α-CD polyrotaxane cross-linking agent with degradable characteristics, and the preparation process is shown in Figure 9. Carbonyldiimidazole (CDI) allowed the hydroxyl groups of the α-CD to be modified by *n*-butyl groups. Each one of α-CD contained 13 *n*-butyl groups, and the substitution of hydroxyl groups of α-CD could be adjusted by controlling the addition of CDI. The *n*-butyl-containing α-CD polyrotaxanes were modified with *2*-aminoethyl methacrylate, so the cross-linkable acrylic acid groups were introduced to the α-CD polyrotaxanes. Light-curing plastics were successfully prepared by copolymerizing α-CD polyrotaxane cross-linking agent with hydroxyethyl methacrylate (HEMA) and urethane (UDMA). Dithiothreitol (DTT) was used to treat the light-curing plastic, resulting in the Vickers hardness of the light-curing plastic decreasing, indicating that an α-CD polyrotaxane cross-linking agent could be used to design and prepare the degradable light-curing plastics. The cleavable plugging groups (disulfide group) were introduced to the extremities of α-CD polyrotaxanes. The cleavage reaction was triggered by the stimulation of DTT, resulting in the structural decomposition of the α-CD polyrotaxanes and the release of α-CD molecules. Furthermore, Seo et al. [38] treated α-CD as the cross-linking agent to react with *n*-butyl and methacrylate to obtain the new resin with a “slide-ring” structure, and then the photodegradable *o*-nitrobenzyl compounds plugged the extremities, as shown in Figure 10. The ultimate tensile strength of the resin decreased to 40% of the initial value with 254 nm UV irradiated.

Drawing on the method of Imran and Harada, our group reported a simple preparation of polyrotaxane cross-linking agent based on α-CD [39,40,41,42]. First, to introduce carbon-carbon double bonds into α-CD, α-CD was modified with C_3_H_5_Br or itaconic anhydride. Then, the hydroxyl groups at the ends of PEG were oxidized to carboxylic acids. The modified α-CD was mixed with the modified PEG fully, and then *1*-Adamantanamine was devoted to plug the extremities. The α-CD polyrotaxane cross-linking agent was copolymerized with acrylamide (AM) to obtain body-expanded particles. The swelling and deformation properties tests were carried out to compare the α-CD polyrotaxane cross-linking agent with the conventional cross-linking agent (*N*, *N*′-methylenebisacrylamide). The test results showed that the “slide-ring” structure hydrogel prepared by α-CD polyrotaxane cross-linking agent had greater swelling and better deformation properties than the conventional hydrogel prepared by the conventional cross-linking agent *N*, *N*′-methylenebisacrylamide. The water solubility of α-CD modified by C_3_H_5_Br was lower, whereas the water solubility of α-CD modified by itaconic anhydride was higher because of the introduction of the polar carboxylic acid groups. However, the preparation of the α-CD polyrotaxane cross-linking agent was cumbersome, our group first mixed the α-CD and PEG and then reacted with itaconic anhydride to obtain α-CD polyrotaxane cross-linking agent [43,44]. Both the hydroxyl groups on the α-CD and the extremity of PEG could react with itaconic anhydride, and both carbon–carbon double bonds on the α-CD and PEG could copolymerize with monomers; thus the plugging operations did not need to be carried out, and the process was simplified. Due to the extremities were not plugged, some CDs may slide from PEG and productivity may decrease.

## 3. The Preparation and Application of Hydrogel Based on β-CD Cross-Linking Agent

**Figure 11:**
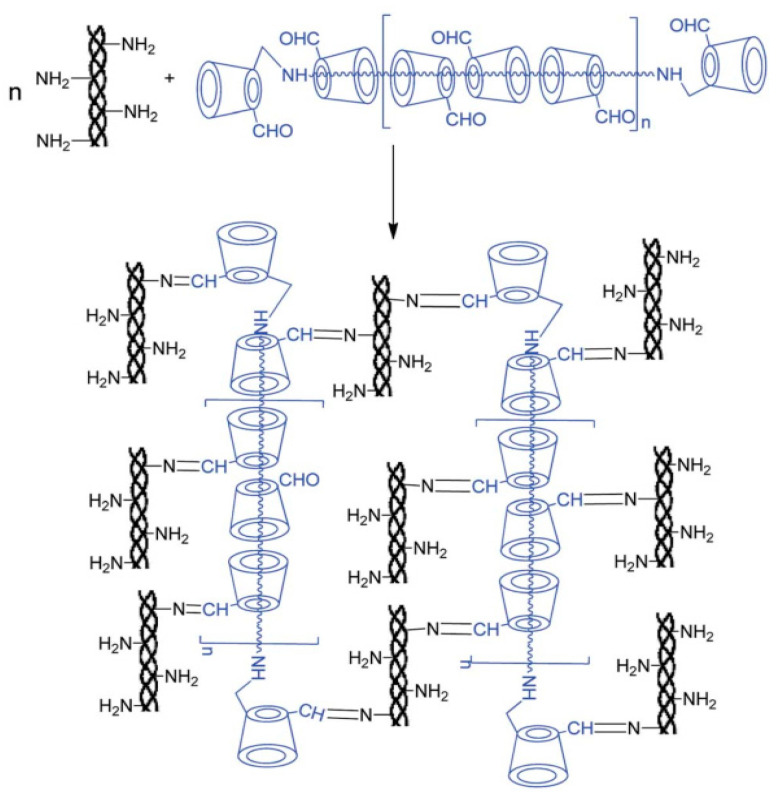
Scheme illustration for the reaction of collagen with β-CD polyrotaxane-CHO [45].

Currently, the synthetic methods regarding α-CD polyrotaxane cross-linking agents were more mature, whereas there were fewer effective synthetic methods for β-CD polyrotaxane cross-linking agents. The cavity size of CD may cause the difference. The cavity of α-CD is smaller, so there are more choices of plugging agents, and, theoretically, as long as the volume of the plugging agent is larger than the cavity of α-CD, α-CD can be prevented from slipping off the molecule chain of the guest molecule. However, the size of the cavity of β-CD was 0.62 nm, and there were fewer types of plugging agents available for it. Liu et al. [45] reported a method based on a biological cross-linking agent (β-CD polyrotaxane polyaldehyde) to prepare a new hydrogel with high biodegradability, specific surface area, compressive modulus, and whose swelling properties are well suited for cell adhesion, as shown in Figure 11. β-CD reacted with polyetheramine (PPG-NH_2_), and then β-CD monoaldehyde was devoted to plug the extremities of β-CD pseudopolyrotaxane to obtain β-CD polyrotaxane. After reduction in β-CD polyrotaxane with NaBH_4_, it was oxidized to the β-CD polyrotaxane polyaldehyde using *2*-iodoylbenzoic acid (IBX) as oxidant and dimethyl sulfoxide as the solvent. Collagen copolymerized with β-CD polyrotaxane polyaldehyde, *N*-(*3*-Dimethylaminopropyl)-*N*′-ethylcarbodiimide hydrochloride (EDC), and glutaraldehyde (GA), respectively, to obtain hydrogel material. Zhao et al. [46] utilized β-CD polyrotaxane polyaldehyde as the cross-linking agent to copolymerize with collagen to prepare a corneal repair material (Col-β-CD-PR) with a tensile strength value of 2.38 ± 0.13 MPa, which was very close to that of a normal human cornea (approximately 3 MPa).

Kubota et al. [47] mixed PPG and β-CD sufficiently and then plugged the extremities. TEMPO and PhI(OAcTf) were added to the mixture to react with the hydroxyl group and generate the aldehyde group. After a full reaction, the PRβCD1 crosslinking agent was obtained. Conventional cross-linking agent GA and the PRβCD1 crosslinking agent were used to copolymerize with monomer to generate Col-GA and Col-PRβCD1. From the stress–strain curves, the fracture stress of the Col-GA significantly increased from 60 ± 6 to 480 ± 65 kPa compared to Col; however, the fracture strain decreased from 40 ± 5% to 20 ± 3.5%. The fracture stress of the Col-PRβCD1 increased about 5.3 times, the fracture strain increased about 1.6 times, and the toughness increased from about 10 kJ/m^3^ to about 90 kJ/m^3^ compared to Col.

**Figure 12:**
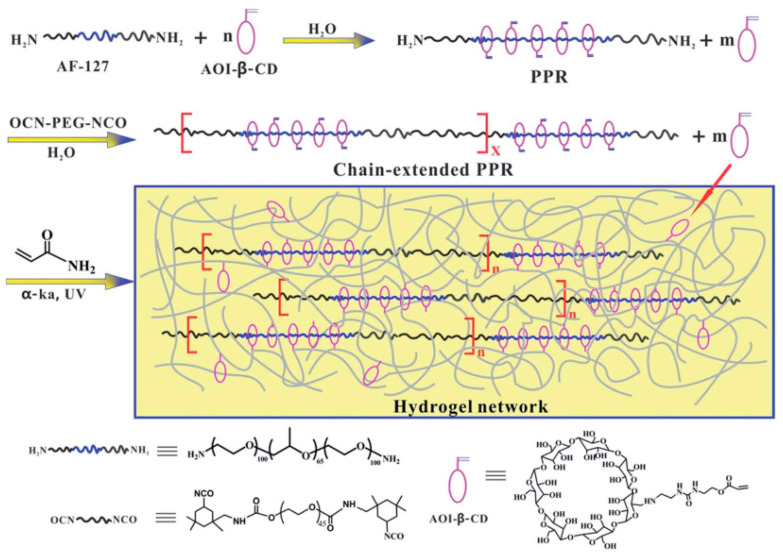
Strain hardening and highly resilient hydrogels crosslinked by chain-extended reactive pseudopolyrotaxane [48].

Cui et al. [48] used *2*-isocyanatoethyl acrylate (AOI) to modify β-CD. PEG-PPG-PEG was added to the mixture and stirred fully to make sure the long-chain molecule passed through the cavity of β-CD. Then, isocyanate groups were employed to plug the extremities of PEG to obtain a new hydrogel, as shown in Figure 12. The hydrogel stretched from 10 to more than 26 times its original length and withstood 95% or even 98% of the compressive strain without breaking.

Nakahata et al. [49,50,51] prepared supramolecular gels with self-healing functions based on β-CD, ferrocene-modified polyacrylic acid crosslinked into a mesh structure.

Our group dissolved β-CD with polyetheramine (PPG-NH_2_) in deionized water and stirred the solution at room temperature [52]. The white precipitate was polyetheramine/β-CD pseudopolyrotaxane. Then, the product was reacted with maleic anhydride in trichloromethane solution by refluxing for 10~15 h in a thermostatic water bath at 60~65 °C. After the reaction was completed, the reactants were cooled to room temperature, and the white precipitate was precipitated and dried to obtain a polyetheramine/β-CD/maleic anhydride polyrotaxane cross-linking agent. Both the hydroxyl groups on β-CD and the amino groups on the extremities of β-CD pseudopolyrotaxane could react with maleic anhydride, resulting in the introduction of carbon–carbon double bonds on the molecule, and β-CD polyrotaxane cross-linking agents were prepared successfully. Compared with other disclosed technical methods, this method could effectively increase the number of carbon–carbon double bonds, and reactivity, and decrease the cost. The swelling property and deformation property tests were carried out to compare the difference of hydrogels based on β-CD polyrotaxane cross-linking agent and *N*, *N*-Methylenebisacrylamide separately copolymerized with AM. The results showed that the β-CD polyrotaxane cross-linking agent hydrogel had a better swelling property and deformation property. As the cost of β-CD is lower than α-CD, the use of β-CD instead of α-CD for the preparation of polyrotaxane cross-linking agents could effectively reduce the cost. However, due to the fact that the extremities were not being plugged in this method, it might cause the β-CD to fall out from the long-chain molecule, resulting in a decrease in productivity and an increase in the difficulty of purification.

## 4. The Preparation and Application of Hydrogel Based on γ-CD Cross-Linking Agent

**Figure 13:**
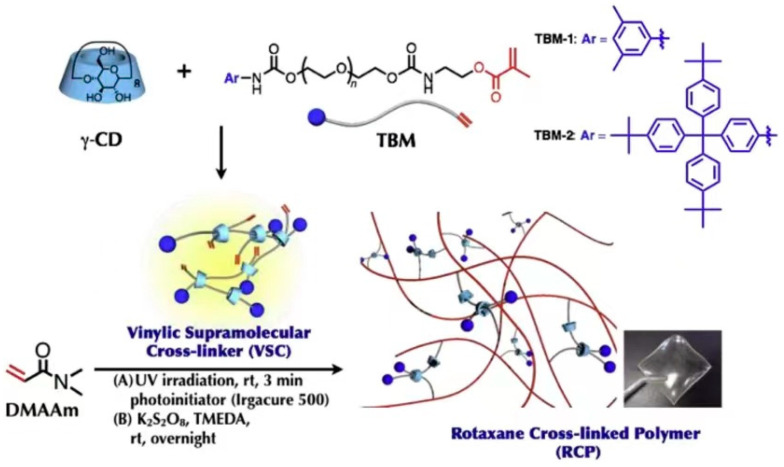
Synthesis of rotaxane cross-linked polymers using *N*, *N*-dimethylacrylamide and rotaxane cross-linking agent [54].

**Figure 14:**
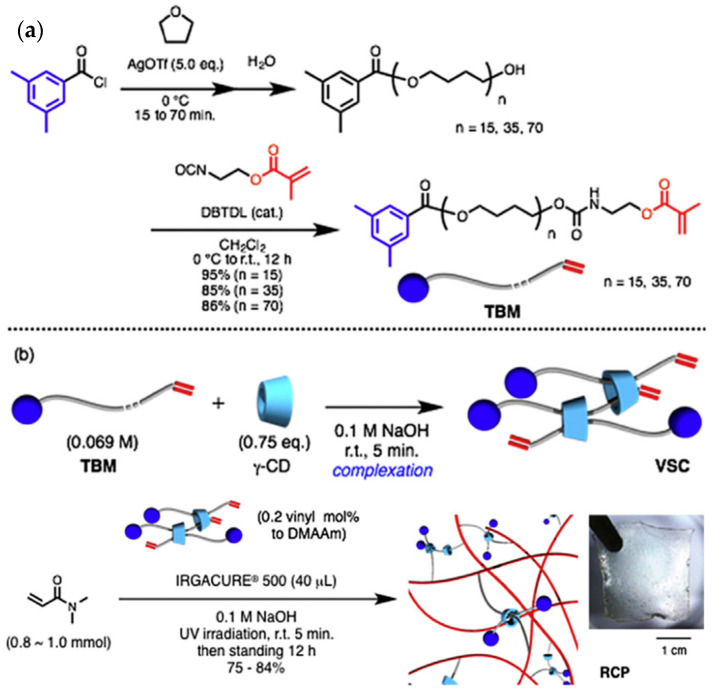
(**a**,**b**) Preparation of rotaxane cross-linked polymers based on γ-CD [55].

Compared with α-CD and β-CD, γ-CD has the biggest cavity size. It may cause the two chain molecules to pass through one cavity or the solution molecules to enter the cavity, which give the material properties different from those of α-CD and β-CD [53]. Jang et al. [54] reported that γ-CD was used to prepare the polyrotaxane cross-linking agent. The extremities of the long-chain molecule were plugged with terminal bulky end tethering PEG macromonomer (TBM) or *4*-(Tris(*4*-tertbutylphenyl) methyl) phenyl isocyanate, as shown in Figure 13. Mix TBM dissolved on polytetrahydrofuran (PTHF) with γ-CD in NaOH solvent and react fully to obtain a γ-CD vinyl supramolecule cross-linking agent (VSC). Then, copolymerize VSC with *N*, *N*-Dimethylacrylamide (DMAAm) to obtain a polyrotaxane cross-linked polymer (RCP) based on γ-CD. Comparing the swelling property with a conventionally cross-linked polymer (CCP) based on PEGDA cross-linking agent, it was found that the swelling rate increased from 500% to 1800% in water and from 1000% to 2400% in DMF solution. According to the results of viscoelastic property at 15% water content, all the SeS curves of two kinds of hydrogels showed yielding and plastic deformation, which indicated the typical glassy polymer films. The CCP had a pressure peak of 5 MPa, and the RCP reached a peak of 27 MPa. Furthermore, the effect of the length of PTHF on γ-CD polyrotaxane polymer was investigated, and the results showed that γ-CD polyrotaxane polymers made from VSCs with longer PTHF chains were more ductile, indicating that the movement range of the cross-linking points directly affected the mechanical properties of the γ-CD polyrotaxane polymer. Compared with the covalent crosslinked polymer, the γ-CD polyrotaxane polymer had a better swelling property. Subsequently, Iijima et al. [55] prepared TBM by cationic ring-opening polymerization of tetrahydrofuran with *3*,*5*-dimethylbenzoyl chloride (initiator and plugging agent), silver trifluoromethanesulfonate, and isocyanate methacrylate. γ-CD was reacted with TBM to obtain VSC, and then VSC was copolymerized with DMAAm to obtain a γ-CD polyrotaxane polymer, as shown in Figure 14.

Malucelli et al. [56] reported that AM was reacted with γ-CD for the introduction of a carbon–carbon double bond. Then, modified γ-CD was copolymerized with *N*-isopropylacrylamide (NIPAAm) to obtain hydrogel. Compared with conventional chemical hydrogel, the new hydrogel had a better swelling property. With an increasing addition of modified γ-CD, the swelling rate decreased gradually. Cosola et al. [57] reported that γ-CD was modified with bis(acyl)phosphaneoxide (BAPO) to generate a new initiator (BAPO-γ-CyD) with the function of cross-linking. It reacted with monomers (AM, etc.) to generate polymer materials with a three-dimensional structure. The polymer material showed a great swelling property. Furthermore, the strength of the polymer material could be adjusted by controlling the addition of BAPO-γ-CyD. Meng et al. [58] added crystalline micro-nanoparticles (CMNPs) into the system of γ-CD and PEG modified with CS. The mixture was copolymerized with PAM to obtain CS-PEG/PAM/γ-CD. When fully reacted, CS-PEG/PAM/γ-CD was sunk in Na_3_Cit solution to obtain the target product CS-PEG/PAM/γ-CD/Cit^3−^. According to the controlled experiments, it could be found that, on the one hand, the properties of CS-PEG/PAM/γ-CD were similar to PAM hydrogel. On the other hand, due to the effects of inter-ionic interactions, the modulus of CS-PEG/PAM/γ-CD/Cit^3−^ increased 58.2 times, the fracture stress increased 13.2 times, and the toughness increased 16.2 times at least. With the addition of CS-PEG, all the strength properties increased.

Our group reported a one-step method for the preparation of a γ-CD cross-linking agent [59]. PEGDA passed through the cavity of γ-CD. The terminal carbon–carbon double bond of γ-CD copolymerized with AM to plug the extremities of the long chain molecules. Compared with the conventional chemical cross-linking agent (MBA), under the condition of 0.5% cross-linking agent addition, the swelling ratio of slide-ring hydrogel reached 79.05 in water, 7.35~9.47 in CaCl_2_ solution, and 8.92~12.02 in NaCl solution, which were much higher than those of conventional hydrogel (8.8). Due to the radius of Ca^2+^ being larger than that of Na^+^, the complexation of the metal ion with the hydroxyl group hindered the sliding of cyclodextrins, thus exhibiting a lower swelling performance in CaCl_2_ solution.

## 5. Conclusions and Outlook

(1) The conventional hydrogel has poor deformability due to the fixed cross-linking points, which limits the application of gel materials. However, polyrotaxane cross-linking agents with a “slide ring” structure can overcome the disadvantage and show high deformability and high recovery properties, which can effectively expand the application fields of gel materials.
(2) At present, the preparation methods of polyrotaxane cross-linking agents based on α-CD are more mature than β-CD and γ-CD. However, some factors also limit the use of the cross-linking agent. On the one hand, the expensive price of α-CD limits its application, so the cheaper β-CD has become a potential material for development. On the other hand, it is necessary to find a convenient and simple method to prepare polyrotaxane cross-linking agent to avoid the cumbersome preparation process. Furthermore, some CD may fall out from the long-chain molecule during the preparation process, which may affect the properties of the hydrogel.
(3) The single chain of the guest molecule passes through the cavity of α-CD or β-CD, whereas the situation that two chains pass through the cavity may occur in γ-CD. The difference makes γ-CD polyrotaxane cross-linking agents have some special preparation methods and properties.
(4) Due to the unique structures of CD polyrotaxane cross-linking agents, researchers should actively seek effective and low-cost synthesis methods and explore the application. In the future, the development and application of CD polyrotaxane cross-linking agents will be more diversified and in-depth.

## Data Availability

The raw/processed data required to reproduce these findings cannot be shared at this time, as the data also form part of an ongoing study.
